# Real-World Analysis of Post-Progression Treatment Patterns and Outcomes for *EGFR* Mutation-Positive Patients Treated with First-Line Osimertinib

**DOI:** 10.3390/curroncol31050182

**Published:** 2024-04-26

**Authors:** Amanda Jane Williams Gibson, Michelle Liane Dean, Ishjot Litt, Adrian Box, Winson Y. Cheung, Vishal Navani

**Affiliations:** 1 Department of Oncology, Cumming School of Medicine, University of Calgary, 3330 Hospital Drive NW, Calgary, AB T2N 4N1, Canada; mlisaac@ucalgary.ca (M.L.D.); ishjot.litt@ucalgary.ca (I.L.); vishal.navani@albertahealthservices.ca (V.N.); 2 Alberta Precision Laboratories, Molecular Pathology Laboratory, 3535 Research Road NW, Calgary, AB T2L 2K8, Canada; adrian.box@albertaprecisionlabs.ca; 3 Division of Medical Oncology, Tom Baker Cancer Centre, Alberta Health Services, 1331 29 St NW, Calgary, AB T2N 4N2, Canada

**Keywords:** *EGFR* mutation-positive, first-line osimertinib, post-progression, real-world, outcomes

## Abstract

**Introduction:** The use of osimertinib in the first-line (1L) setting is an effective treatment option for sensitizing *EGFR*-mutations (*EGFR*m+) and has significantly altered the standard of care practice for *EGFR*m+ disease in Canada. Unfortunately, acquired resistance to osimertinib is almost universal, and outcomes are disparate. Post-progression treatment patterns and the outcome of real-world Canadian *EGFR*m+ patients receiving 1L osimertinib were the focus of this retrospective review. **Methods:** The Glans-Look Lung Cancer Research database was used to identify and collect demographic, clinical, treatment, and outcome data on *EGFR*m+ patients who received 1L osimertinib in the Canadian province of Alberta between 2018 and 2022. **Results:** A total of 150 patients receiving 1L osimertinib were identified. In total, 86 developed progressive disease, with 56 (65%) continuing systemic therapy, 73% continuing osimertinib, and 27% switching to second-line (2L) systemic therapy. Patients were similar both in clinical characteristics at 1L osimertinib initiation and patterns of treatment failure at progression; those continuing 1L osimertinib post-progression had a longer time to progression (13.5 vs. 8.8 months, *p* = 0.05) and subsequent post-osimertinib initiation survival (34.7 vs. 22.8 months, *p* = 0.11). **Conclusions**: The continuation of osimertinib post-progression is an effective disease management strategy for select real-world *EGFR*m+ patients, providing continued clinical benefit, potentially due to different underlying disease pathogenesis.

## 1. Introduction

The discovery of actionable somatic mutations within the epidermal growth factor gene receptor (*EGFR*) challenged the understanding of non-small cell lung cancer (NSCLC) as a single-entity disease. *EGFR* mutations are detected in 10–15% and upwards of 30% of NSCLC diagnoses in Western populations and East Asian populations, respectively [1,2,3]. The subsequent development of precision therapeutics that could target and override the oncogenic action of *EGFR*-mutations has revolutionized the management and prognosis of individuals with *EGFR*-mutation-positive NSCLC (*EGFR*m+) [1,4,5,6,7,8].

Osimertinib, a third-generation *EGFR*-inhibitor, is proficient in targeting both *EGFR*-sensitizing mutations (exon 19 deletions and exon 21 L858R point mutation, which account for around 90% of all detected *EGFR*-mutations) and the T790M resistance mutation [3,9,10]. As a standard of care, first-line (1L) targeted therapy for advanced *EGFR* mutation-positive NSCLC, osimertinib, is an effective and tolerable treatment that improves the prognosis of real-world patients with *EGFR* mutation-positive lung malignancies [11,12].

Unfortunately, mechanisms of resistance to osimertinib-mediated *EGFR*-inhibition arise, often within two years of osimertinib initiation, allowing disease progression to occur [10]. Effective disease management, in the face of *EGFR*-dependent (additional mutations arising within the *EGFR* gene) and/or *EGFR*-independent (non-*EGFR* oncogene activation, amplification, fusion, or histological transformation) resistance to targeted therapy is often unclear. [13]

In Canada, the current consensus is that *EGFR*m+ patients undergo platinum-based chemotherapy or enter an available clinical trial following the termination of 1L osimertinib [14]; however, additional considerations, including comorbidities or patient functional status, determine the viability of other systemic therapy options (osimertinib continued beyond progression, platinum-doublet or single-agent chemotherapy, or clinical trial enrollment). This landscape is rapidly changing, and current clinical trials (e.g., SAVANNAH and MARIPOSA-2) will likely change the recommended post-progression management of *EGFR*m+ disease [15,16,17]. However, the use of osimertinib concurrent with chemotherapy, as per FLAURA2, or the introduction of amivantamab, as per MARIPOSA-1, into the treatment paradigm has yet to receive Health Canada approval. Treatment decisions are further complicated by a current lack of funding for targeted options beyond the 1L setting in Canada.

A lack of clarity regarding the treatment choices that provide the greatest clinical benefit following progression on 1L osimertinib is a current knowledge gap. To help address this unmet clinical need, this study aims to characterize a population of patients receiving first-line osimertinib for advanced *EGFR*-mutation positive NSCLC and describe the post-progression treatment patterns and outcomes of this population.

## 2. Materials and Methods

This study used the Glans-Look Lung Cancer Research (GLR) database, which captures patient-level demographic, clinical, treatment, response, and outcome data by means of chart reviews of electronic medical records for all patients with a diagnosis of lung cancer who receive treatment within the Canadian province of Alberta. GLR data were collected and managed using the Research Electronic Data Capture data capture tool hosted at the University of Calgary [18,19] and under an ongoing institutional review board-approved protocol at our institution (HREBA.CC-16-0574).

### 2.1. Patient Selection

This retrospective cohort included all adult patients with a diagnosis of *EGFR*m+ NSCLC receiving osimertinib as the 1L systemic therapy for advanced disease (unresectable, not amenable to treatment with definitive-intent concurrent chemoradiotherapy, or the presence of metastatic disease). Staging was based on the American Joint Committee on Cancer eighth edition criteria [20].

Testing for lung biomarkers was performed in accordance with the International Association for the Study of Lung Cancer/Association for Molecular Pathology/College of American Pathologists biomarker guidelines [21]. As per these guidelines, patients with advanced NSCLC disease with any component of adenocarcinoma or those with squamous histology without a history of tobacco use, were assessed for *EGFR* alterations, identified in tandem with alterations in other recommended lung cancer biomarker genes (e.g., *KRAS*/*BRAF*/*PIK3CA*) using a DNA-based tumor profiling panel (Agena iPLEX HS Lung Panel) and RNA-based next-generation sequencing assay for relevant lung fusions.

#### Clinical Response and Outcome to Systemic Therapies

Treatment sequence, response, and outcome were calculated using data elements within the GLR.

Treatment response to osimertinib was determined using the Response Evaluation Criteria in Solid Tumors (RECIST) version 1.1 criteria and described using definitions defined within the literature [22]. If diagnostic imaging did not include measurements for retrospective RECIST calculation, response was based on the documented opinion of the reviewing radiologist; this alternate method has been demonstrated to be in good concordance with RECIST calculated best overall response [23].

Treatment post-progression was defined as osimertinib therapy extending for >90 days (approximately three cycles) after the documentation of progressive disease. Rapid progressive disease on osimertinib was defined as progression occurring in the lower quartile of all recorded times to progression. Osimertinib-initiated adverse events were derived from the GLR, which retrospectively identifies the occurrence and management of adverse events through the review of electronic medical records.

Time-to-event endpoints for osimertinib therapy or any subsequent systemic therapy lines were calculated, including the duration of treatment (DoT: the interval between osimertinib initiation and termination), duration of response (DoR: the interval between the date at which the imaging-based best response was recorded until the definitive progressive disease was detected or death occurred during osimertinib therapy), real-world calculations of median PFS (RW-mPFS: the interval from systemic therapy initiation until the detection of definitive progressive disease) [24], and median overall survival measures (the interval following the initiation of osimertinib to death/censoring at the last follow-up (post-osimertinib OS), or the interval between the initial detection of progressive disease on osimertinib to death or censored at last follow-up (post-progression OS).

### 2.2. Statistical Methods

Demographic and clinical characteristics of the study cohort were summarized using descriptive statistics and univariate methods, including Fisher’s exact test for categorical variables, the Kruskal–Wallis test for continuous events and time-to-event models, which were assessed using the Kaplan–Meier approach. A two-sided *p*-value of 0.05 or less was considered a priori as statistically significant. All statistical analyses were performed using Stata Statistics/Data Analysis version 12.1 [25].

## 3. Results

### 3.1. Baseline Characteristics and Response to Osimertinib

A total of 150 *EGFR*mutation-positive patients receiving first-line osimertinib for advanced NSCLC between 2018 and 2022 were identified and followed until death or the data analysis cut-off date (15 August 2023). Demographic and clinical characteristics at 1L osimertinib initiation and the response and outcome on osimertinib are described in Table 1 as follows: Patients were mostly female (62%), had no history of smoking (56%), and possessed an exon 19 deletion (58%). The majority had an ECOG performance status <2 (67%) at the time of osimertinib initiation and one or more sites of metastatic disease (65%). Central nervous system metastases at the time of 1L osimertinib initiation were identified in 23%. Osimertinib elicited a 49% objective response rate and a 17.4-month aggregate PFS within this real-world cohort.

At the time of analysis, 57% of patients were still alive with a median survival time post-osimertinib initiation of 28.9 months.

### 3.2. Post-Osimertinib Treatment Patterns

Eighty-six patients (57% of real-world cohort) experienced progressive disease during osimertinib therapy, 70% of which had progressive disease in the thorax, with or without concurrent distant progression. In total, 8% (n = 7) showed progressive disease in the brain upon initial progression on 1L osimertinib. Twenty-one patients (24%) experienced rapid progression on osimertinib, with a median time to failure of 4.4 months.

**Figure 1:**
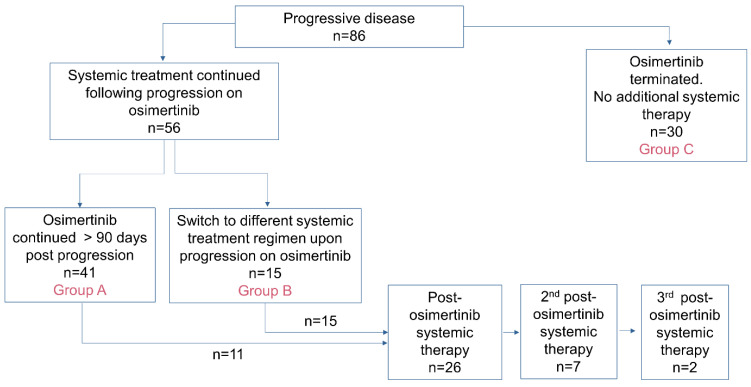
Cohort overview and management decisions upon progression with osimertinib.

Following the detection of progressive disease during osimertinib therapy, three major treatment patterns were observed: (Figure 1)

- Group A (n = 41): upon progression, 1L osimertinib was continued >90 days.
- Group B (n = 15): upon progression, 1L osimertinib was terminated and 2L systemic therapy was initiated.
- Group C (n = 30): upon progression, 1L osimertinib was terminated, and no further systemic therapy was received.

Differences in metastatic burden at osimertinib initiation, the duration of treatment, disease control, metastatic burden at progression, and survival were observed between the three groups (A, B, and C), with the poorest outcomes observed among Group C, those terminating 1L osimertinib and not undergoing any additional systemic therapy (Table 1). Multivariate analysis, when controlling for multiple confounders, did not identify any features prognostic of time to progression (overall model fit *p* = 0.71).

### 3.3. CNS Disease and Non-Systemic Modes of Disease Control

Twenty percent (n = 8) and 33% (n = 5) of patients had CNS-based disease upon osimertinib initiation in Group A and Group B, respectively. Rates of CNS-based disease upon initial progression on 1L osimertinib were also similar among Group A and Group B. Progression of pre-existing CNS disease occurred in 14% (n = 1) of patients in Group A and 40% (n = 2) of patients in Group B.

The use of non-systemic means of disease control differed between Group A and Group B, where the use of thoracic stereotactic body radiotherapy (SBRT) to manage oligometastatic disease was observed in Group A, but not Group B (17% vs. 0%, *p* = 0.03). Stereotactic radiotherapy (SRS) to the brain or surgical resection of CNS-based disease was observed in both Group A and B and did not differ significantly (40% vs. 25%, *p* = 0.42).

### 3.4. Systemic Therapy Regimens Post-Progression

#### 3.4.1. Osimertinib Therapy > 90 Day Post Progression

Among patients continuing 1L osimertinib post-progression (Group A, n = 41), the median time of additional 1L osimertinib therapy was 6.9 months. The majority of Group A patients continued osimertinib at their original starting dose of 80 mg/day (82%), had one or more sites of distant metastases (74%)—most commonly metastases to the bone (61%)—and possessed an EGFR Exon 19 deletion (56%). Of the 10 patients with CNS involvement by the time of progression on 1L osimertinib continuation, 7 (70%) had controlled CNS disease and progressive disease was only observed outside of the CNS. Following a period of >90 days of continued 1L osimertinib post-progression, the subsequent termination of 1L osimertinib was due to treatment failure (66%: 29% death; 37% additional disease progression) (Table 2).

#### 3.4.2. Second-Line Systemic Therapy Regimens Post-Progression

Twenty-six patients received 2L systemic therapy after terminating osimertinib: in addition to the n = 15 patients comprising Group B, n = 11 patients in Group A ultimately terminated osimertinib and initiated 2L systemic therapy.

Second-line systemic therapy was predominantly (65%) platinum-doublet cytotoxic chemotherapy, with the remainder undertaking a rechallenge of osimertinib after an interval of no systemic therapy, receiving a second-generation *EGFR*-inhibitor (gefitinib) or receiving targeted therapies in an investigative setting (osimertinib + savolitinib; tepotinib; platinum-doublet + amivantamab/lazeritinib) (Table 3). The initiation of 2L systemic therapy post-osimertinib progression, whether received immediately upon detection of progression, or following a period of osimertinib use post-progression, elicited a median 7.0 months of disease control.

Seven patients received additional genomic testing following progression on osimertinib, all within the context of clinical trial eligibility: 5 patients were reported to have c-MET amplification and received systemic therapy upon osimertinib termination reflecting this finding (n = 4 SAVANNAH Trial, osimertinib + savolitinib; n = 1 tepotinib).

Univariate analysis revealed that among those patients receiving 2L systemic therapy (n = 26), no statistically significant difference in post-progression OS was observed between those making an immediate switch to a 2L systemic therapy regimen (Group B) versus the continuation of 1L osimertinib >90 days post-progression and then switching to 2L systemic therapy (n = 11 within Group A) (18 months vs. 11.8 months, log-rank *p* = 0.23). Similarly, for those continuing osimertinib > 90 days post-progression (Group A), no statistically significant difference in post-progression OS was observed between those with and without 2L systemic therapy (18.0 vs. 11.0 months, log-rank *p* = 0.37).

### 3.5. Treatment Strategy and Outcome

At 1L osimertinib initiation, Group B had a higher proportion of ≥3 unique metastatic sites than Group A (22% vs. 47%, *p* = 0.008) but otherwise did not differ in terms of demographic or clinical characteristics. At the time of initial progression on 1L osimertinib, Group A and Group B were clinically similar in relation to the nature of 1L osimertinib failure but differed in time-to-event endpoints, where Group A had longer RW-mPFS compared to Group B (13.5 vs. 8.8 months, log-rank *p* = 0.05), and a numerically longer, but statistically insignificant post-osimertinib OS (34.7 vs. 22.8 months, log-rank *p* = 0.11). Multivariate analysis, controlling for multiple confounders, identified males at increased risk of a shorter post-progression survival (HR: 2.2, [95%CI: 1.1–4.6]; *p* = 0.04).

## 4. Discussion

This study was a retrospective review of 150 *EGFR*m+ patients treated with 1L osimertinib therapy, according to standard of care practice in Canada. With a focus on the treatment decision and outcome of 86 patients with documented progressive disease on 1L osimertinib, this study plays a role in quantifying the disease management decisions made in real-world clinical settings upon progression on 1L osimertinib. This addresses a current knowledge gap in the management of *EGFR*m+ disease, as no additional targeted treatment options are currently approved by health regulatory agencies.

This study was able to capture the diversity of real-world patients *EGFR*m+ treated with 1L osimertinib, reporting on treatment response, post-progression treatment patterns, and outcome using a real-world population-level cohort managed within the Canadian province of Alberta. When compared to the FLAURA clinical trial cohort, this study cohort had a lower proportion of patients of Asian ancestry, included patients with ECOG performance status ≥2 [10], and had both shorter median exposure to osimertinib treatment (15.6 vs. 20.7 months) and overall survival (28.9 vs. 38.6 months) [26]. This real-world study cohort more closely resembles other real-world 1L osimertinib-treated *EGFR*m+ cohorts, exhibiting similar durations of disease control on 1L osimertinib (median PFS 17.4 months vs. 16.2–22.0 months) [11,27,28].

Unique to this study was the identification and examination of the treatment choices, patterns, and outcomes of patients who were fit for treatment and able to continue systemic therapy post-progression on 1L osimertinib. This study was able to compare the notable majority who continued 1L osimertinib treatment > 90 days post-progression (Group A median duration of post-progression osimertinib: 6.9 months) and those who transitioned to 2L therapy within 90 days of the detection of progressive disease on 1L osimertinib (Group B) [Table 1]. Group A and B differed significantly in the time to progression on 1L osimertinib (13.5 vs. 8.8 months, log-rank *p* = 0.05) and the subsequent use of stereotactic body radiotherapy (SBRT) for control of thoracic oligoprogressive disease (17% vs. 0%, *p* = 0.03). Given similar patient characteristics at 1L osimertinib initiation, comparable disease control and tolerability to 1L osimertinib, and similar patterns of progressive disease on 1L osimertinib—particularly in the context of analogous incidence and control of CNS-based disease—suggests the potential of a more indolent disease process operating in Group A compared to Group B. The significantly different speed at which progressive disease appears and/or the development of different manifestations of osimertinib-resistant progressive disease—which may or may not be amenable to SBRT—facilitates the continued use of 1L osimertinib with ongoing clinical benefit beyond progression in some patients. More rapid failure, increasing liver involvement (13% at osimertinib initiation, 40% at progression), and the inability to manage thoracic progression via stereotactic body radiation were observed among those discontinuing 1L osimertinib and switching to 2L therapy upon progression (Group B); these factors may influence clinical decision making upon progression, and are again suggestive of a different pathogenesis operating between Group A and Group B. Indeed, those with disease amenable to the continued use of 1L osimertinib >90 days post-progression had statistically insignificant, but numerically longer survival time from osimertinib initiation than those who switched to 2L therapy (34.7 vs. 22.8 months, *p* = 0.11), which is a clinically meaningful survival difference in the context of advanced/metastatic NSCLC where survival times are limited. While the proportion of patients developing intracranial disease upon progression on 1L osimertinib did not differ significantly between those patients who continued 1L osimertinib and those who transitioned to an alternate 2L therapy, among those continuing 1L osimertinib post-progression, a high level of intracranial disease control was observed (70%) [Table 2]. This, in addition to the other factors enumerated above, may also influence clinical decision making, given the paucity of other therapies with effective CNS penetration/control in the context of EGFRm+ NSCLC.

Second-line therapy rates for this study cohort were higher than other 1L osimertinib-treated *EGFR*m+ cohorts (31% vs. 13%) [29] but similarly relied on platinum-doublet regimens, eliciting a median of 7 months of disease control in this study cohort. This may also hold importance in the decision to continue 1L osimertinib or transition to alternate 2L systemic therapies, where the use of cytotoxic treatment regimens may not be acceptable (comorbidities/performance status and/or patient preference). Third-line systemic therapy was used by a small proportion (14%) of the cohort, but the presence of declining performance status (43% ECOG ≥ 2) and a high burden of metastatic disease (70% M1c) likely impacted outcome and may have precluded subsequent lines of systemic therapy. [30] This supports previous findings suggesting that treatment choice within the 1L setting is most impactful on outcome. Adequate disease control and optimization of patient performance protect the ability of patients to receive 2L systemic therapy [31], which is currently limited to clinical trials or cytotoxic platinum-based therapies, both of which are predicated on good patient functional status, the latter of which requires a number of weeks for therapy to translate into tumor response. Indeed, the ability to receive both osimertinib post-progression and a subsequent 2L therapy was associated with an extended survival time in a distinct, albeit statistically insignificant manner: receiving both osimertinib >90 days post-progression and subsequent 2L systemic therapy resulted in an additional 6 months of post-progression survival when compared to osimertinib received post-progression but without 2L systemic therapy (18 vs. 11 months, log-rank *p* = 0.37), or an immediate switch to 2L systemic therapy (18 vs. 11.8 months, log-rank *p* = 0.23).

**Figure 2:**
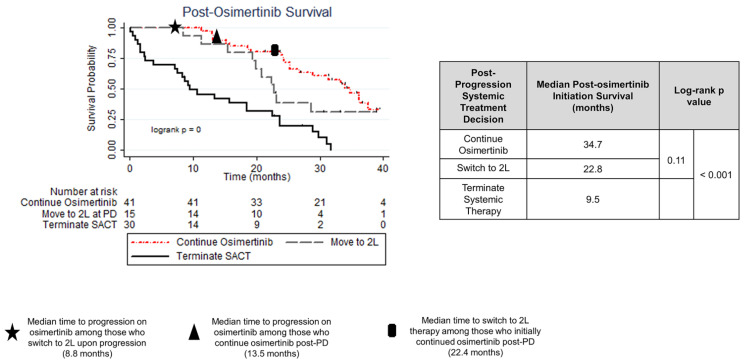
Post-osimertinib initiation survival by systemic treatment decisions upon progression on 1L osimertinib.

Importantly, this study also identifies a need for additional tools to guide the decision-making process and help identify the underlying differences between patients who may appear similar clinically before and during 1L therapy but then exhibit different outcomes. A high incidence of c*MET* amplification (71% of all tested individuals, with testing only received in relation to clinical trial enrollment and not as a standard of care) was identified upon progression on 1L osimertinib via clinical trial-funded genomic testing, coupled with the identification of a group of patients who exhibited relatively rapid failure and death during 1L osimertinib (Group C: median progression-free survival: 8.6 months, post-osimertinib initiation OS: 9.5 months; Figure 2). This indicates a significant unmet need for a precision oncology lens to be applied to *EGFR*m+ disease, such as a standard of care repeat or expanded molecular testing for *EGFR*m+ patients. An improved understanding of factors impacting disease pathogenesis alongside the identification of possible mechanisms of primary or acquired resistance to systemic therapy, either molecular (i.e., *BRAF*, *MET*, *HER2*) [32,33] or histologic (small-cell transformation, present in 3–10% of *EGFR*m+ and squamous cell carcinoma transformation, present in up to 15% of patients treated with targeted therapy) [34,35], would help guide treatment and disease management, which optimize patient outcome and quality of life.

This study possesses important limitations **as follows**: as a retrospective, real-world review, there is a lack of consistency in standardized response assessment and patient follow-up schedules, and the improved outcomes seen in the context of *EGFR*m+ disease treated with 1L osimertinib means that outcome data will continue to mature. Furthermore, treatment selection biases are inherent in real-world decision making with respect to post-progression management with both systemic and non-systemic therapies. It is challenging to delineate the cause and effect given these inherent biases, and statistical approaches to minimize these biases by “matching” patient populations were not undertaken.

Despite these acknowledged limitations, this study does have some particular strengths. Foremost, this is a real-world population-based study and, therefore, represents all *EGFR*m+ patients treated with 1L osimertinib in routine clinical practice within the Canadian province of Alberta (population of ~4.6 million) [36]. Second, Canada possesses a single-payer universal healthcare model and is, thus, representative of other jurisdictions with socialized healthcare provision. This method of healthcare delivery provides equal treatment coverage irrespective of financial situation or healthcare provider, and within this study, eliminates potential biases in cohort identification. Information regarding best practice for management of *EGFR*m+ disease upon development of progressive disease scarce in the real-world context, and inclusive and comprehensive real-world datasets that can address this knowledge gap are rare. The Glans-Look Research database represents one of North America’s largest real-world evidence databases containing the type of data that are crucial to understanding the effectiveness of treatment decisions and outcomes of patients treated in the real-world clinical setting.

In summary, to our knowledge, this retrospective real-world study of Canadian *EGFR*m+ patients treated with 1L osimertinib represents the first population-level review of the treatment outcomes and patterns of patient post-progression on 1L osimertinib. Importantly, this study confirms that in the context of delayed progression, oligometastatic disease amenable to control by SBRT, treatment tolerability, and lack of available targeted 2L options, the continued use of osimertinib post-progression may continue to provide clinical benefit for some patients, particularly if the initiation of 2L therapy can be delayed until clinically indicated. An improved understanding of the underlying mechanisms of resistance to osimertinib would aid in guiding post-progression treatment choices and decisions, and serve to further increase outcomes in *EGFR*m+ disease.

## Figures and Tables

**Table 1:** Characteristics and response to osimertinib in the study cohort by group.

Characteristic	Entire Cohort (n = 150)	Progressive Disease Noted during 1L Osimertinib Therapy (n = 86)		
		Osimertinib ≥ 90 Days Post-PD Group A (n = 41)	2L Systemic Therapy at PD Group B (n = 15)	No additional Systemic Therapy at PD Group C (n = 30)
**Sex**				
Female Male	93 (62) 57 (38)	24 (58) 17 (42)	9 (60) 6 (40)	22 (73) 8 (27)
**Smoking history**				
Ever Never	66 (44) 84 (56)	22 (54) 19 (46)	8 (53) 7 (47)	18 (60) 12 (40)
**Age (years)**				
Median (IQR)	68.3 (61.3–78.4)	64.3 (54.9–77.6)	69.2 (59.4–75.3)	71.5 (62.4–80.4)
**Asian ancestry**				
No Yes	104 (69) 46 (31)	25 (61) 16 (49)	12 (80) 3 (20)	19 (63) 11 (37)
**EGFR mutation**				
Exon 19 deletion Exon 21^L858R^	87 (58) 63 (42)	23 (56) 18 (44)	5 (33) 10 (67)	17 (57) 13 (43)
**ECOG at 1L osimertinib initiation**				
ECOG < 2 ECOG ≥ 2	100 (67) 50 (33)	34 (83) 7 (17)	12 (80) 3 (20)	10 (33) 20 (67)
**TNM 8th Edition M-stage at 1L osimertinib initiation**				
M0 M1a M1b M1c	16 (11) 37 (24) 48 (32) 49 (33)	5 (13) 10 (24) 16 (39) 10 (24)	0 (0) 4 (26) 4 (26) 7 (48)	2 (7) 6 (20) 10 (33) 12 (40)
**≥3 sites of metastatic disease at 1L osimertinib initiation**				
No Yes	112 (81) 29 (19)	36 (88) 5 (12)	8 (54) 7 (46) *	24 (60) 6 (20)
**Metastatic disease in liver, bone or CNS present upon 1L osimertinib initiation**				
Liver Bone Central Nervous System	28 (19) 72 (48) 35 (23)	7 (17) 19 (46) 8 (20)	2 (13) 10 (67) 5 (33)	10 (67) * 15 (100) * 6 (40)
**Concurrent mutations** **(known at time of osimertinib initiation)**				
None BRAF KRAS PIK3CA ERBB2	141 (94) 1 (<1) 1 (<1) 7 (5) 0 (0)	40 (98) 1 (2) 0 (0) 0 (0) 0 (0)	15 0 (0) 0 (0) 0 (0) 0 (0)	26 (87) 0 (0) 1 (3) 3 (10) 0 (0)
**Duration of treatment (months)** Median (IQR)	15.6 (6.0–26.9)	26.8 * (18.7–33.8)	9.4 (6.9–16.0)	8.0 (2.7–25.7)
**Tolerability**				
Adverse events—no intervention required	71%	83%	67%	57%
**Toxicity**				
Adverse events—intervention required (dose reduction, treatment break, hospitalization, treatment termination)	35%	29%	40%	17%
**Real-world objective response rate (rwORR)**	49%	54%	60%	56%
**Real-world disease control rate (rwDCR)**	82%	100%	100%	76% *
**Primary resistance**	5%	0%	0%	3%
**Time to progression**				
Median [95% confidence interval]	17.4 [14.7–23.3]	13.5 [10.0–17.2]	8.8 [5.6–11.9]	8.6 [5.8–16.2]
**Nature of progression**				
Unknown (rapid clinical decline) Thoracic progression only Distant progression only Thoracic + distant progression	-	0 (0) 22 (54) 11 (27) 8 (19)	0 (0) 6 (40) 3 (20) 6 (40)	15 (50) 6 (20) 3 (10) 6 (20)
**Metastatic disease in liver, bone or CNS present upon progression**				
Liver Bone Central Nervous System	-	7 (17) 25 (61) 10 (24)	6 (40) 10 (67) 5 (33)	4 (13) 17 (57) 5 (17)
**≥3 Unique sites of metastatic disease upon progression**				
No Yes	-	29 (71) 12 (29)	7 (46) 8 (53)	22 (73) 8 (27)
**Development or progression of CNS disease**				
No Yes	-	38 (93) 3 (7)	13 (7) 2 (13)	29 (97) 1 (3)
**Survival post-progression on with osimertinib**				
Median [95% Confidence interval]	-	14.9 [8.7–24.9]	11.8 [4.9–17.0]	1.9 * [0.9–3.7]
**Survival following osimertinib initiation**				
Median [95% Confidence interval]	28.9 [25.9–NR]	34.7 [25.2–45.9]	22.8 [15.4–NR]	9.5 * [7.1–18.5]
**3-year survival rate**	27%	46%	33%	0% *

ECOG: Eastern Cooperative Oncology Group; IQR: interquartile range; CNS: central nervous system; NR: not reached; * denotes significance at α = 0.05.

**Table 2:** Osimertinib post-progression cohort: characteristics at time of 1L osimertinib continuation and treatment response.

Characteristic	Osimertinib ≥ 90 Days Post-PD Group A (n = 41) n (%)
**Sex**	
Female Male	24 (58) 17 (42)
**Age (years) at 1L osimertinib continuation**	
Median (IQR)	65.1 (56.4–78.1)
**Asian ancestry**	
No Yes	25 (61) 16 (39)
**EGFR mutation**	
Exon 19 deletion Exon 21^L858R^	23 (56) 18 (44)
**TNM 8th Edition M-stage at 1L osimertinib continuation**	
M0 M1a M1b M1c	4 (10) 11 (27) 11 (27) 15 (36)
**≥3 sites of metastatic disease**	
No Yes	29 (71) 12 (29)
**Metastatic disease in liver, bone or CNS present upon 1L osimertinib continuation**	
Liver Bone Central Nervous System	7 (17) 25 (61) 10 (24)
**CNS present (but controlled) at 1L osimertinib continuation**	7/10 (70%)
**Osimertinib continued on reduced dose**	
No Yes (50% reduction to 40mg/day)	37 (82) 7 (17)
**Adverse events post-progression (requiring intervention)**	
Treatment break (Grade 2 Fatigue; 14 days)	1 (2)
**Duration of treatment post-progression (months)**	
Median (IQR)	6.9 (4.3–14.3)
**Reason for osimertinib termination**	
Toxicity Death New treatment identified Further progressive disease Osimertinib ongoing	1 (2) 12 (29) 1 (2) 15 (37) 12 (29)
**Second-Line (non-osimertinib) therapy received**	11 (38)

ECOG: Eastern Cooperative Oncology Group; IQR: interquartile range; CNS: central nervous system.

**Table 3:** Characteristics, systemic therapy regimen and outcomes of patients opting for alternate systemic therapy following 1L osimertinib by treatment line.

Clinical Measure	By Post-Osimertinib Systemic Therapy Line		
	2L n (%) (n = 26)	3L n (%) (n = 7)	4L n (%) (n = 2)
***Cytotoxic chemotherapy***	***17 (65)***	***6 (86)***	***0 (0)***
Platinum-doublet NSCLC regimen Single-agent regimen	(n = 17) (n = 0)	(n = 0) (n = 6)	
***Immune checkpoint inhibitor***	***0 (0)***	***0 (0)***	***1 (50)***
***Targeted therapy***	***7 (27)***	***1 (14)***	***1 (50)***
Second generation EGFR TKI Osimertinib rechallenge after treatment break SAVANNAH (osimertinib + savolitinib) trial Tepotinib	(n = 2) (n = 1) (n = 4) (n = 0)	(n = 0) (n = 1) (n = 0) (n = 0)	(n = 0) (n = 0) (n = 0) (n = 1)
***Cytotoxic chemotherapy and targeted therapy***	2 (8)	0 (0)	0 (0)
Platinum-doublet + osimertinib MARIPOSA-2 trial (Arm C: platinum doublet + amivantamab + lazertinib)	(n = 1) (n = 1)		
**ECOG at initiation**			
ECOG < 2 ECOG ≥ 2	19 (73) 7 (27)	4 (57) 3 (43)	1 (50) 1 (50)
**M-stage at initiation**			
M0 M1a M1b M1c	1 (4) 4 (15) 7 (27) 14 (54)	0 (0) 1 (15) 1 (15) 5 (70)	0 (0) 0 (0) 0 (0) 2 (100)
**Duration of treatment (months)** Median (IQR)	3.7 (1.4–11.4)	1.8 (0–4.2)	3.8 (2.5–5.1)
**Real-world progression-free survival (months)** Median [95% confidence interval]	7.0 [5.2–9.0]	5.0 [0.7–NR]	NR
**Nature of initial progression**	(n = 18)	(n = 6)	(n = 2)
Thoracic progression only Distant progression only Both thoracic and distant progression	7 (39) 3 (17) 8 (44)	2 (33) 2 (33) 2 (33)	0 (0) 1 (50) 1 (50)
**Progressive disease in the brain upon initial progression**	(n = 18)	(n = 6)	(n = 2)
No Yes	9 (50) 9 (50)	6 (100) 0 (0)	1 (50) 1 (50)
**Reason for treatment termination**			
Treatment ongoing Completed as planned Progressive disease ECOG decline/death Adverse events	6 (23) 1 (3) 12 (44) 4 (15) 4 (15)	1 (14) 1 (14) 4 (58) 1 (14) 0 (0)	1 (50) 0 (0) 0 (0) 1 (50) 0 (0)

## Data Availability

The data presented in this study are not publicly available due to ethics restrictions on the sharing of data.
